# Association Between Irritable Bowel Syndrome and Hypothyroidism: Insights from Large-Scale Population-Based Studies

**DOI:** 10.2174/0118715303390590250928210823

**Published:** 2025-11-24

**Authors:** Jing Wu, Ziwei Liu, Luna Liu, Qian Liu, Shiwei Sun, Huijie Li, Fei Li, Meng Zhou, Yongfeng Song

**Affiliations:** 1 Key Laboratory of Endocrine Glucose & Lipids Metabolism and Brain Aging, Ministry of Education, Department of Endocrinology, Shandong Provincial Hospital Affiliated to Shandong First Medical University, Jinan, Shandong, 250021, China;; 2 Shandong Clinical Research Center of Diabetes and Metabolic Diseases, Jinan, Shandong, 250021, China;; 3 Shandong Institute of Endocrine and Metabolic Diseases, Jinan, Shandong, 250021, China;; 4 “Chuangxin China” Innovation Base of Stem Cell and Gene Therapy for Endocrine Metabolic Diseases, Jinan, Shandong, 250021, China;; 5 Shandong Engineering Laboratory of Prevention and Control for Endocrine and Metabolic Diseases, Jinan, Shandong, 250021, China;; 6 Shandong Engineering Research Center of Stem Cell and Gene Therapy for Endocrine and Metabolic Diseases, Jinan, Shandong, 250021, China;; 7 Department of Statistics and Medical Records Management, Shandong Provincial Hospital Affiliated to Shandong First Medical University, Jinan, Shandong, 250021, China;; 8 Shandong Provincial Hospital, Shandong University of Traditional Chinese Medicine, Jinan, Shandong, 250021, China;; 9 Department of Endocrinology, Central Hospital Affiliated to Shandong First Medical University, Jinan, Shandong, 250013, China

**Keywords:** Irritable bowel syndrome, hypothyroidism, retrospective cohort, cross-sectional analysis, risk association, propensity score matching

## Abstract

**Introduction:**

Irritable Bowel Syndrome (IBS) and hypothyroidism are both common conditions that significantly affect patient health. This study examines the link between IBS and hypothyroidism, focusing on how IBS impacts hypothyroidism.

**Methods:**

A retrospective cohort study using data from the UK Biobank (UKB) and a cross-sectional analysis from the National Inpatient Sample (NIS) were conducted. Propensity score matching was applied to control for confounding factors. Cox proportional hazards models (UKB) and logistic regression models (NIS) were used to evaluate the association between IBS and hypothyroidism. Subgroup analyses by age and sex were performed.

**Results:**

The UKB cohort included 22,970 IBS patients (mean age 56.1 ± 7.94 years, 72.2% female) with a hypothyroidism prevalence of 4.1%, compared to 438,094 non-IBS participants (mean age 56.4 ± 8.12 years, 51.9% female) with a prevalence of 2.9%. In the NIS, 183,738 IBS patients had a hypothyroidism prevalence of 20.6%, compared to 10.3% in 20,298,589 non-IBS participants. After PSM, the hazard ratio (HR) for hypothyroidism in IBS patients was 1.21 (95% CI: 1.12–1.30, *P* < 0.001) in the UKB, and the odds ratio (OR) was 1.25 (95% CI: 1.23–1.27, *P* < 0.001) in the NIS. Subgroup analyses showed a higher risk for hypothyroidism in IBS patients, particularly those aged ≤65 years and females.

**Conclusion:**

IBS is associated with an increased risk of hypothyroidism. Clinicians should consider screening for thyroid dysfunction in IBS patients to improve patient outcomes.

## 1 INTRODUCTION

Irritable bowel syndrome (IBS) is a multifactorial gastrointestinal disorder characterized by chronic abdominal pain associated with a change in the frequency or form of stool, and influenced by diverse etiological factors including genetic and epigenetic factors, gut microbiota disorders, inflammation, increased abnormal colonic motility or transit, disruption of brain-gut mutual communication, altered gut physiology interactions, and psychosocial factors [1]. Its symptoms, which include abdominal pain, bloating, and altered bowel habits, can severely impact quality of life [2]. Recent studies have indicated a potential association between IBS and autoimmune conditions such as hypothyroidism, a disorder that affects 4-10% of the global population, resulting in insufficient thyroid hormones and can lead to various metabolic disturbances [3, 4]. Given the rising global prevalence of both conditions, it is essential to explore their potential relationship further to enhance patient management and develop targeted interventions. Understanding this association could provide insights into shared pathophysiological mechanisms, ultimately benefiting clinical management strategies and patient outcomes.

The motivation for conducting this research stems from the recognition that while IBS and hypothyroidism are often studied independently, the interplay between them remains largely unexplored [5, 6]. Previous literature has indicated that individuals with autoimmune conditions may have higher incidences of gastrointestinal issues, yet there is limited empirical data evaluating the specific association between IBS and hypothyroidism [7-9]. This gap in the literature calls for more comprehensive investigations to establish whether these two conditions are merely coincidental comorbidities or if there is a more profound, interrelated pathophysiological mechanism at play. Identifying any correlations can inform healthcare providers about the potential need for comprehensive screening in patients presenting with either condition. Additionally, understanding demographic and lifestyle factors that influence this association can offer a more nuanced view of patient management strategies. This study aims to clarify the association between IBS and hypothyroidism, utilizing comprehensive data to analyze demographic and clinical variables involved. By delineating the relationship between these conditions, we hope to enhance clinical awareness and inform effective management strategies for patients suffering from both IBS and hypothyroidism, ultimately improving their quality of life.

## 2 MATERIALS AND METHODS

### 2.1 Data Sources

Study samples were collected from the UK Biobank (UKB) and the National Inpatient Sample (NIS) Database. Briefly, the UKB recruited over 500,000 participants aged 40 to 69 years from 2006 to 2010, with detailed information available elsewhere [10]. The NIS is the most extensive publicly available healthcare database in the United States and was developed by the Healthcare Cost and Utilization Project (HCUP). This database comprises approximately a 20% stratified sample of all discharges from U.S. community hospitals. UKB's extensive phenotyping enables exploration of genetic susceptibility loci and microbiome interactions, while NIS's population-level hospitalization data permits assessment of disease co-occurrence patterns and healthcare burden. In this study, our analysis of the NIS utilized data from 2016 to 2018.

The UKB study received approval from the North West Multi-Centre Research Ethics Committee (Ref: 11/NW/0382). All participants provided signed consent for participation electronically. The NIS was exempt from approval by the Institutional Review Board because it uses previously collected de-identified data.

### 2.2 Study Design

We examined the association between the incidence of hypothyroidism and IBS. Study participants were divided into two groups based on the presence or absence of IBS. All participants with no missing data on age, sex, race, and lifestyle factors (smoking and drinking) were included in this study. For the analyses conducted using the UKB, we excluded participants with prevalent hypothyroidism at baseline. The flowchart of the selection of the study population is present in Fig. (**1**). As a result, our final analytical sample comprised 461,064 participants from UKB and 20,482,327 participants from the NIS.

The International Classification of Disease-10^th^ Revision (ICD-10) codes and the International Classification of Disease-10^th^ Revision-Clinical Modification (ICD-10-CM) codes were used to diagnose participants with IBS and hypothyroidism in the UKB and NIS, respectively. We also attained the following variables: age, sex, race, smoking status, alcohol consumption, obesity, glucose status, hypertension, and dyslipidemia. The ICD codes used to identify these diagnoses were listed in Supplementary Table **1**.

### 2.3 Statistical Analysis

Demographic data for both groups were analyzed using Student's t-test and the x^2^ test for continuous and categorical data, respectively. Follow-up of participants in UKB began at baseline and ended when the relevant outcome was available or October 31, 2022, whichever came first. We constructed 3 models to adjust for baseline covariates. Model 1 was designed to adjust for age and sex. Model 2 included each factor in model 1 and further considered race, smoking status, and drinking status. Model 3 incorporated all variables from model 2 and additionally accounted for obesity, glucose status, hypertension, and dyslipidemia. To explore heterogeneity within IBS subtypes, we further stratified IBS patients into IBS with diarrhea (K580), IBS with constipation (K581), and other subtypes (K582, K588, K589) based on ICD-10 codes. The associations between these subtypes and hypothyroidism were analyzed using multivariable logistic regression in the NIS cohort.

In the UKB, we used Cox proportional hazards regression to determine longitudinal associations between IBS and hypothyroidism outcomes. We plotted Kaplan-Meier (KM) curves for the occurrence of hypothyroidism based on the presence or absence of IBS. In the NIS, logistic regression models are used to examine the association between IBS and hypothyroidism by estimating odds ratios (ORs) and their corresponding 95% confidence intervals (CIs). The Bonferroni test was used to take a *P*-value less than 0.003 (16 tests) as statistically significant. Propensity score matching (PSM) was conducted using the nearest neighbor method with a 1:3 matching ratio, ensuring a balanced comparison between the groups. The balance of covariates after matching was assessed using standardized mean differences (SMDs), with SMD values <0.10 indicating adequate balance. The propensity score took into account factors including age, gender, race, smoking status, drinking, obesity, glucose status, hypertension, and dyslipidemia. To examine subpopulations susceptible to population-related differences, subgroup analyses by sex and age were performed in both databases. *P-*values <0.05 were considered statistically significant. All statistical analyses were performed using R (version 4.2.2).

## 3 RESULTS

### 3.1 Characteristics of the Study Population

Table **1** outlines the baseline characteristics of participants in the UKB and the NIS before and after PSM. After PSM, all covariates achieved excellent balance, with standardized mean differences (SMDs) <0.10 for both cohorts (Supplementary Table **2**). Among the UKB cohort, there were 22,970 participants with IBS and 438,094 without IBS. Significant difference in age (mean 56.1 *vs*. 56.4 years, *P* < 0.001) and sex (female comprising 72.2% *vs*. 51.9%, *P* < 0.001) was noted. Additionally, a higher prevalence of smoking was observed in IBS patients (44.1% *vs*. 45.2%, *P* =0.001), and they were less likely to drink alcohol (76.6% *vs*. 81.3%, *P* < 0.001). The rates of obesity were elevated in IBS patients at 23.9% compared to 24.2% among those without IBS (*P* < 0.001). More concerning was the higher incidence of dyslipidemia (3.6% *vs*. 3.2%, *P* < 0.001) among IBS patients, which may exacerbate their health issues. In the NIS, 183,738 participants had IBS, while 20,298,589 did not. A substantial shift towards older ages was evident among IBS patients compared to non-IBS participants (mean 61.0 *vs*. 49.9 years, *P* <0.001), while male representation in the IBS group was only 19.1% compared to 43.8% in the non-IBS group (*P* < 0.001). Furthermore, IBS patients exhibited significantly higher rates of obesity (19.4% *vs*. 13.3%, *P* < 0.001), hypertension (60.0% *vs*. 46.0%, *P* < 0.001), and dyslipidemia (41.2% *vs*. 27.7%, *P* < 0.001). These findings emphasize the complex characteristics associated with IBS and highlight the necessity for understanding how these variables may influence the potential connection to hypothyroidism, the primary focus of our research.

### 3.2 Associations between IBS and Hypothyroidism

**Fig. (2):**
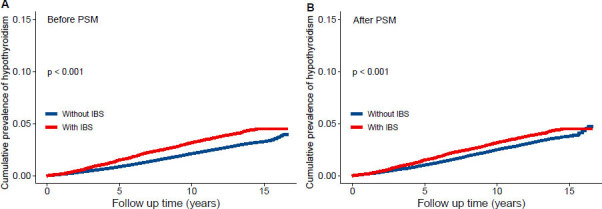
KM curves for the occurrence of hypothyroidism in the UKB. (**A**) Before PSM. (**B**) After PSM. **Abbreviations**: KM, Kaplan-Meier; UKB, UK Biobank; PSM, Propensity score matching; IBS, Irritable bowel syndrome.

Table **2** presents the associations between IBS and hypothyroidism in both the UKB and the NIS. In the UKB, with a median follow-up time of 13.7 years, the HR for hypothyroidism in participants with IBS showed a significant increase across various models. In Model 1, adjusted for age and sex, the HR was 1.22 (95% CI: 1.14, 1.31, *P* < 0.001). This association persisted in Model 2, which included additional adjustments for race, smoking status, and drinking status, yielding an HR of 1.20 (95% CI: 1.12, 1.29, *P* < 0.001). After further adjustments for obesity, glucose status, hypertension, and dyslipidemia in Model 3, the HR remained significant at 1.19 (95% CI: 1.11, 1.27, *P* < 0.001). Moreover, when employing PSM, the HR was 1.21 (95% CI: 1.12, 1.30, *P* < 0.001), reinforcing the connection between IBS and hypothyroidism. Similarly, the NIS data supported a strong association, with the OR for hypothyroidism in IBS patients showing a consistent trend. In Model 1, adjusted for age and sex, the OR was 1.51 (95% CI: 1.49, 1.53, *P* < 0.001), and this association remained significant in Model 2 (OR: 1.40, 95% CI: 1.39, 1.42, *P* < 0.001) and Model 3 (OR: 1.36, 95% CI: 1.35, 1.38, *P* < 0.001), which included adjustments for additional demographic and clinical factors. After PSM, the OR was 1.25 (95% CI: 1.23, 1.27, *P* < 0.001). The KM curves further illustrated the cumulative incidence of hypothyroidism in the UKB based on IBS status. There was a statistically significant difference in the cumulative occurrence of hypothyroidism before and after PSM (Fig. **2**). Stratified analyses of IBS subtypes in the NIS revealed significant associations across all subtypes (Supplementary Table **3**).

### 3.3 Subgroup Analysis of the Association between IBS and Hypothyroidism

**Fig. (3):**
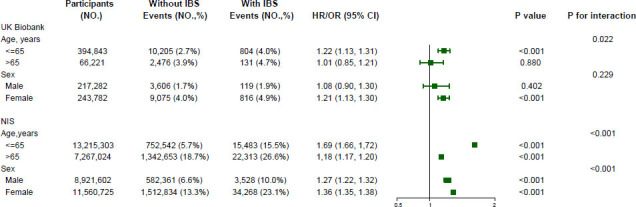
Subgroup analyses of the association of IBS and hypothyroidism. Each subgroup was adjusted for age, sex, race, smoking status, drinking status, obesity, glucose status, hypertension, and dyslipidemia. **Abbreviations**: IBS, irritable bowel syndrome; HR, hazard ratio; OR, odds ratio; CI, confidence interval; NIS, National Inpatient Sample.

Subgroup analyses from both the UKB and the NIS reveal important associations between IBS and hypothyroidism. In the UKB, participants aged ≤65 with IBS demonstrated a significantly higher incidence of hypothyroidism (4.0%) compared to those without IBS (2.7%), yielding a hazard ratio (HR) of 1.22 (95% CI: 1.13, 1.31, *P* < 0.001). However, among participants aged >65, no significant association was observed (HR: 1.01, 95% CI: 0.85, 1.21, *P* = 0.880), suggesting possible age-related heterogeneity. No statistically significant interaction between the effects of irritable bowel syndrome and hypothyroidism by sex (*P* for interaction ≥ 0.05). Analyses from the NIS highlighted a much stronger association; in younger participants aged ≤65, IBS patients had a significantly increased hypothyroidism rate of 15.5% compared to 5.7% without IBS, with an OR of 1.69 (95% CI: 1.66, 1.72, *P* < 0.001). Among those aged >65, the rate was also elevated (26.6% *vs*. 18.7%) with an OR of 1.18 (95% CI: 1.17, 1.20, *P* < 0.001). Both female (23.1% *vs*. 13.3%, OR: 1.36, 95% CI: 1.35, 1.38, *P* < 0.001) and male (10.0% *vs*. 6.6%, OR: 1.27, 95% CI: 1.22, 1.32, *P* < 0.001) IBS patients in the NIS cohort similarly exhibited higher risks (Fig. **3**). Collectively, these findings underline the variability in the association between IBS and hypothyroidism across different datasets, highlighting the necessity for targeted screening and management strategies.

## 4 DISCUSSION

### 4.1 Principal Findings

This study identified a significant association between IBS and hypothyroidism using two large, independent cohorts: the UKB and the NIS. Individuals with IBS were found to have an increased risk of hypothyroidism, with this association remaining consistent across multiple statistical models, including those adjusting for demographic, clinical, and lifestyle factors. The findings were robust in subgroup analyses across sex and age groups and persisted after PSM, underscoring the reliability of the results. These observations suggest a potential link between IBS and hypothyroidism that merits further investigation.

### 4.2 Comparison with Prior Evidence and Potential Mechanisms

Importantly, existing evidence more commonly suggests that thyroid dysfunction affects bowel habits (*e.g*., through altered motility or gut-brain axis disruption) rather than IBS directly causing hypothyroidism. Our findings do not establish causality, and we have critically evaluated the directionality, including the possibility of shared underlying mechanisms such as autoimmunity (*e.g*., Hashimoto’s thyroiditis) or bidirectional influences mediated by chronic inflammation and immune dysregulation.

This study extends prior research that has primarily explored IBS and hypothyroidism as distinct clinical entities [11-13]. Although previous studies have reported a high prevalence of gastrointestinal symptoms in autoimmune thyroid disorders and suggested that thyroid dysfunction may influence bowel habits, this investigation is among the first to comprehensively evaluate the relationship between IBS and hypothyroidism in large, population-based datasets [4, 14, 15]. Compared to smaller, single-center studies or analyses of selected populations, the findings from this study are bolstered by the scale and diversity of the datasets, which enhance the generalizability of the results.

Several plausible mechanisms may explain the observed association. Chronic low-grade inflammation, a hallmark of both IBS and autoimmune thyroid disease, may provide a shared pathophysiological basis [16, 17]. Hypothyroidism disrupts inflammatory balance, metabolism, and neuroendocrine signaling, potentially exacerbating IBS [18]. Thyroid deficiency elevates pro-inflammatory cytokines (*e.g*., IL-6, TNF-α) and suppresses anti-inflammatory IL-10, worsening gut inflammation and visceral hypersensitivity. It also impairs lipid/carbohydrate metabolism, reducing mitochondrial function and promoting insulin resistance, which may alter gut motility and microbial fermentation [19-21]. Reduced thyroid hormones down-regulate serotonin transporters, diminishing gut secretory reflexes and amplifying pain perception [22, 23]. Additionally, autoimmune links such as Hashimoto’s thyroiditis suggest shared triggers, while thyroid dysfunction may enhance immune responses *via* dendritic cell activation and T-cell dysregulation, increasing risks of autoimmune-driven gastrointestinal pathologies [20, 24, 25]. Hypothyroidism is also associated with alterations in gastrointestinal motility, including delayed gastric emptying and prolonged intestinal transit time, which can mimic or exacerbate IBS symptoms [18-20, 24]. Additionally, disruptions in the gut-brain axis and gut microbiota have been implicated in both conditions, potentially linking IBS to autoimmune thyroid disorders *via* immune dysregulation or microbial imbalance [16, 26, 27]. Importantly, autoimmune thyroiditis, such as Hashimoto’s thyroiditis, is often observed in patients with IBS-like gastrointestinal symptoms, which further supports the idea of a shared immune-mediated pathway between the two conditions. Hormonal changes associated with hypothyroidism may further contribute to IBS symptoms by affecting gut motility, increasing visceral sensitivity, and altering gut-brain communication [28]. These mechanisms highlight the complexity of the relationship between hypothyroidism and IBS and underscore the need for further research, including biomarker studies and microbiome analyses, to identify the exact causal pathways that link these two conditions.

### 4.3 Strengths and Limitations

This study has several strengths. While the use of two large, independent cohorts—one population-based (UKB) and one hospital-based (NIS)—provides complementary evidence for the association between IBS and hypothyroidism, we acknowledge that comparing these datasets in parallel may introduce challenges. Specifically, the NIS, as a hospital-based dataset, is prone to ascertainment and selection bias (*e.g*., over-representation of sicker individuals with multiple comorbidities), which may limit the external validity of NIS findings for community populations. The comprehensive adjustment for demographic, clinical, and lifestyle factors, along with the application of PSM, minimizes confounding and strengthens the reliability of the findings [29]. The inclusion of subgroup analyses further elucidates the consistency of the association across age and sex groups, adding granularity to the results. However, certain limitations must be acknowledged. The observational design precludes causal inferences, leaving the directionality of the association unclear. The reliance on ICD codes for diagnosing IBS and hypothyroidism may introduce misclassification bias, as subclinical hypothyroidism and functional bowel disorders other than IBS may not be accurately distinguished [30-34]. Residual confounding from unmeasured factors such as physical activity, socioeconomic status, psychological stress, anxiety, depression, and dietary patterns—all highly relevant to both IBS and thyroid disorders—is possible, as these were not adjusted for in our analysis due to data constraints in the UKB and NIS datasets. For instance, in the UKB cohort, while dietary data are available through questionnaires, their granularity and potential recall bias may limit adjustments for these confounders. The NIS database, lacking detailed dietary or autoimmune markers, further restricts the ability to disentangle these effects. Moreover, the cross-sectional nature of the NIS data limits the ability to explore temporal relationships. Besides, the NIS primarily captures individuals requiring hospitalization, which may introduce selection bias (*e.g.*, over-representation of patients with commodities or advanced disease stages). This could inflate the observed prevalence of hypothyroidism in IBS patients compared to the general population. Despite these limitations, the consistency of the findings across two large and diverse datasets strengthens confidence in the observed associations.

### 4.4 Clinical Implications and Conclusions

These findings underscore the importance of considering thyroid dysfunction in patients with IBS and vice versa. Screening for thyroid dysfunction in patients with IBS may be considered, but a cost-benefit analysis is needed to guide clinical practice. Future research should prioritize elucidating the causal pathways linking IBS and hypothyroidism, including the roles of inflammation, autoimmunity, and gut microbiota. Longitudinal studies and interventional trials will be crucial to determine whether early screening and management of thyroid dysfunction in IBS patients can mitigate symptom severity and improve quality of life. By advancing our understanding of this association, these findings provide a foundation for more integrated, multidisciplinary care approaches for patients with coexisting gastrointestinal and endocrine conditions.

## AUTHORS’ CONTRIBUTIONS

The authors confirm their cintribution to the paper as follows: JW: Data Analysis and Interpretation. ZL: Writing-Original Draft Preparation. LL: Data Collection. QL: Visualization. SS: Investigation. HL: Conceptualization. FL: Writing -Reviewing and Editing. MZ: Validation. YF: Study Concept or Design. All authors read and approved the final version submitted for publication.

## Figures and Tables

**Fig. (1):**
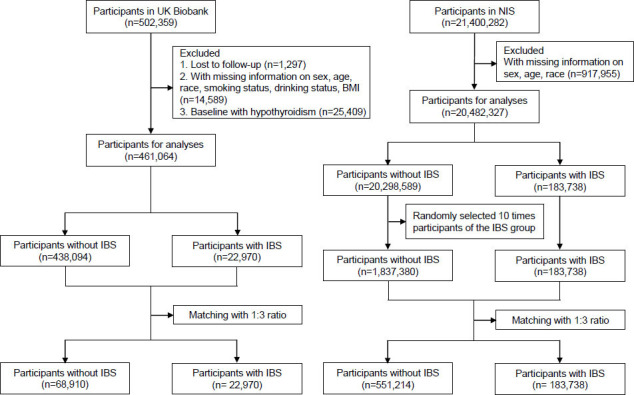
Flowchart of the selection of the study population from the UKB and NIS. **Abbreviation**: UKB, UK Biobank; NIS, National Inpatient Sample; IBS, Irritable bowel syndrome.

**Table 1:** Baseline characteristics of the study population.

**Characteristic**	**UK Biobank Before PSM**		***P* Value**	**UK Biobank After PSM**		***P* Value**	**NIS Before PSM**		***P* Value**	**NIS After PSM**		***P* Value**
	**Without IBS** **(n =438,094)**	**With IBS** **(n =22,970)**		**Without IBS** **(n =68,910)**	**With IBS** **(n =22,970)**		**Without IBS** **(n = 20,298,589)**	**With IBS** **(n =183,738)**		**Without IBS** **(n = 551,214)**	**With IBS** **(n =183,738)**	
Age, mean (SD)	56.4 (8.12)	56.1 (7.94)	<0.001	56.1 (7.94)	56.1 (7.94)	0.800	49.9 (27.2)	61.0 (18.1)	<0.001	61.4 (18.2)	61.0 (18.1)	<0.001
Gender (%)	-	-	-	-	-	-	-	-	-	-	-	-
Male	210,886 (48.1%)	6,396 (27.8%)	<0.001	19,201 (27.9%)	6396 (27.8%)	0.963	8,886,425 (43.8%)	35,177 (19.1%)	<0.001	105,794 (19.2%)	35,177 (19.1%)	0.655
Female	227,208 (51.9%)	16,574 (72.2%)	-	49,709 (72.1%)	16,574 (72.2%)	-	11,412,164 (56.2%)	148,561 (80.9%)	-	445,420 (80.8%)	148,561 (80.9%)	-
Race (%)	-	-	-	-	-	-	-	-	-	-	-	-
White	414,165 (94.5%)	22,193 (96.6%)	<0.001	66,609 (96.7%)	22,193 (96.6%)	0.767	13,148,360 (64.8%)	156,154 (85.0%)	<0.001	468,172 (84.9%)	156,154 (85.0%)	0.588
Other	23,929 (5.5%)	777 (3.4%)	-	2301 (3.3%)	777 (3.4%)	-	7,150,229 (35.2%)	27,584 (15.0%)	-	83,042 (15.1%)	27,584 (15.0%)	-
Smoking (%)	197,941 (45.2%)	10,132 (44.1%)	0.001	30,403 (44.1%)	10,132 (44.1%)	0.985	6,044,612 (29.8%)	68,914 (37.5%)	<0.001	208,424 (37.8%)	68,914 (37.5%)	0.020
Drinking (%)	356,221 (81.3%)	17,584 (76.6%)	<0.001	52,762 (76.6%)	17,584 (76.6%)	0.971	1,011,801 (5.0%)	6,770 (3.7%)	<0.001	21,150 (3.8%)	6,770 (3.7%)	0.003
Obesity (%)	106,127 (24.2%)	5,483 (23.9%)	0.225	16,485 (23.9%)	5,483 (23.9%)	0.879	2,691,209 (13.3%)	35,647 (19.4%)	<0.001	106,563 (19.3%)	35,647 (19.4%)	0.522
Glucose status (%)	-	-	-	-	-	-	-	-	-	-	-	-
Normal	429,630 (98.1%)	22,530 (98.1%)	0.825	67,656 (98.2%)	22,530 (98.1%)	0.340	15,531,108 (76.5%)	136,819 (74.5%)	<0.001	406,889 (73.8%)	136,819 (74.5%)	<0.001
Prediabetes	124 (0.0%)	8 (0.0%)	-	14 (0.0%)	8 (0.0%)	-	144,848 (0.7%)	2,604 (1.4%)	-	6,052 (1.1%)	2,604 (1.4%)	-
Diabetes	8,340 (1.9%)	432 (1.9%)	-	1,240 (1.8%)	432 (1.9%)	-	4,622,633 (22.8%)	44,315 (24.1%)	-	138,273 (25.1%)	44,315 (24.1%)	-
Hypertension (%)	32,420 (7.4%)	2,047 (8.9%)	<0.001	6,210 (9.0%)	2,047 (8.9%)	0.655	9,337,080 (46.0%)	110,223 (60.0%)	<0.001	332,737 (60.4%)	110,223 (60.0%)	0.004
Dyslipidemia (%)	13,913 (3.2%)	829 (3.6%)	<0.001	2,397 (3.5%)	829 (3.6%)	0.362	5,623,896 (27.7%)	75,700 (41.2%)	<0.001	221,272 (40.1%)	75,700 (41.2%)	<0.001

**Table 2:** Association between IBS and hypothyroidism in the UK Biobank and NIS.

-	**UK Biobank**				**NIS**			
	**Without IBS** **Events (NO., %)**	**With IBS** **Events (NO., %)**	**HR (95% CI)**	***P* Value**	**Without IBS** **Events (NO., %)**	**With IBS** **Events (NO., %)**	**OR (95% CI)**	***P* Value**
Model 1^a^	12,681 (2.9%)	935 (4.1%)	1.22 (1.14, 1.31)	<0.001	2,095,195 (10.3%)	37,796 (20.6%)	1.51 (1.49, 1.53)	<0.001
Model 2^b^			1.20 (1.12, 1.29)	<0.001			1.40 (1.39, 1.42)	<0.001
Model 3^c^			1.19 (1.11, 1.27)	<0.001			1.36 (1.35, 1.38)	<0.001
PSM	2327 (3.4%)	935 (4.1%)	1.21 (1.12, 1.30)	<0.001	94,522 (17.1%)	37,796 (20.6%)	1.25 (1.23, 1.27)	<0.001

**Note:** ^a^Model 1 was adjusted for age and sex.

^b^Model 2 was adjusted for model 1 plus race, smoking status, drinking status.

^c^Model 3 was adjusted for model 2 plus obesity, glucose status, hypertension, dyslipidemia.

**Abbreviations:** NIS, National Inpatient Sample; HR, hazard ratio; OR, odds ratio; CI confidence interval.

## Data Availability

The data supporting the study's findings are included in the article. For additional inquiries, please contact the corresponding author or refer to the supplementary materials. Data comes from the UK Biobank (application number 89483) can contact access@ukbiobank.ac.uk for details. Data from the NIS can be obtained from the corresponding author upon reasonable request..

## LIST OF ABBREVIATIONS

IBS: Irritable Bowel Syndrome
UKB: UK Biobank
NIS: National Inpatient Sample
PSM: Propensity Score Matching
HR: Hazard Ratio
OR: Odds Ratio
CI: Confidence Interval
HCUP: Healthcare Cost and Utilization Project
ICD-10-CM: International Classification of Disease-10^th^ Revision-Clinical Modification
KM: Kaplan-Meier

## STANDARDS OF REPORTING

STROBE guidelines were followed.
