# Supplementary material for: Association Between Irritable Bowel Syndrome and Hypothyroidism: Insights from Large-Scale Population-Based Studies
Source: Endocr Metab Immune Disord Drug Targets. 2025 Nov 24;26:E18715303390590. doi: 10.2174/0118715303390590250928210823 (PMC13284642; doi:10.2174/0118715303390590250928210823)
Supplement: Supplementary file 1 [file EMIDDT-26-E18715303390590_SD1.pdf]

Supplementary Material

Association Between Irritable Bowel Syndrome and Hypothyroidism: Insights from Large-Scale Population-Based Studies

Jing Wu[1,2,3,4,5,6,#], Ziwei Liu[1,2,3,4,5,6,#], Luna Liu[1,2,3,4,5,6,#], Qian Liu[1,2,3,4,5,6,#], Shiwei Sun[1,2,3,4,5,6], Huijie Li[7,8,*], Fei Li[1,2,3,4,5,6,*], Meng Zhou[1,2,3,4,5,6,*] and Yongfeng Song[1,2,3,4,5,6,9,*]

[1]Key Laboratory of Endocrine Glucose & Lipids Metabolism and Brain Aging, Ministry of Education, Department of Endocrinology, Shandong Provincial Hospital Affiliated to Shandong First Medical University, Jinan, Shandong, 250021, China; [2]Shandong Clinical Research Center of Diabetes and Metabolic Diseases, Jinan, Shandong, 250021, China; [3]Shandong Institute of Endocrine and Metabolic Diseases, Jinan, Shandong, 250021, China; [4]"Chuangxin China" Innovation Base of Stem Cell and Gene Therapy for Endocrine Metabolic Diseases, Jinan, Shandong, 250021, China; [5]Shandong Engineering Laboratory of Prevention and Control for Endocrine and Metabolic Diseases, Jinan, Shandong, 250021, China; [6]Shandong Engineering Research Center of Stem Cell and Gene Therapy for Endocrine and Metabolic Diseases, Jinan, Shandong, 250021, China; [7]Department of Statistics and Medical Records Management, Shandong Provincial Hospital Affiliated to Shandong First Medical University, Jinan, Shandong, 250021, China; [8]Shandong Provincial Hospital, Shandong University of Traditional Chinese Medicine, Jinan, Shandong, 250021, China; [9]Department of Endocrinology, Central Hospital Affiliated to Shandong First Medical University, Jinan, Shandong, 250013, China

Supplementary Table 1 ICD codes used to identify diagnosis.

**Diagnosis**	**ICD-10 codes (UK Biobank)**	**ICD-10-CM codes (NIS)**
IBS	K58	K58
Hypothyroidism	E02, E03	E02, E03
Smoking	Identified by touchscreen	Z87891, F172
Drinking	Identified by touchscreen	F101, F102
Obesity	Diagnosed by BMI	E660-E662, E668, E669
Hypertension	I10-I13, I15	I10-I13, I15, I16
Prediabetes	R730	R730
Diabetes	E10, E11	E10, E11
Dyslipidemia	E780-E786	E780-E786

Supplementary Table 1 SMD values for each variable after PSM.

SMD	UK Biobank	NIS
Age	0.020	0.002
Gender	0.001	<0.001
Race	0.001	0.002
Smoking	0.006	<0.001
Drinking	0.008	<0.001
Obesity	0.002	0.001
Glucose status	0.036	0.011
Hypertension	0.008	0.004
Dyslipidemia	0.022	0.007

STROBE Statement—checklist of items that should be included in reports of observational studies

	Item No.	Recommendation	Page No.	Relevant text from manuscript
**Title and abstract**	1	(*a*) Indicate the study's design with a commonly used term in the title or the abstract	1-2	Line 1-2
		(*b*) Provide in the abstract an informative and balanced summary of what was done and what was found	2	Line 26-28
**Introduction**				
Background/rationale	2	Explain the scientific background and rationale for the investigation being reported	3	Line 40-60
Objectives	3	State specific objectives, including any prespecified hypotheses	4	Line 61-71
**Methods**				
Study design	4	Present key elements of study design early in the paper	5	Line 92-99
Setting	5	Describe the setting, locations, and relevant dates, including periods of recruitment, exposure, follow-up, and data collection	4-5	Line 75-84
Participants	6	a) ***Cohort study***—Give the eligibility criteria, and the sources and methods of selection of participants. Describe methods of follow-up<br>b) ***Case-control study***—Give the eligibility criteria, and the sources and methods of case ascertainment and control selection. Give the rationale for the choice of cases and controls<br>c) ***Cross-sectional study***—Give the eligibility criteria, and the sources and methods of selection of participants	5	Line 97-99
		d) ***Cohort study***—For matched studies, give matching criteria and number of exposed and unexposed<br>*e)* ***Case-control study***—For matched studies, give matching criteria and the number of controls per case	5	Line 95-97
Variables	7	Clearly define all outcomes, exposures, predictors, potential confounders, and effect modifiers. Give diagnostic criteria, if applicable	5-6	Line 104-106
Data sources/ measurement	8*	For each variable of interest, give sources of data and details of methods of assessment (measurement). Describe comparability of assessment		

		methods if there is more than one group		
Bias	9	Describe any efforts to address potential sources of bias	6	Line 109-118
Study size	10	Explain how the study size was arrived at	5	Line 97-99

Quantitative variables	11	Explain how quantitative variables were handled in the analyses. If applicable, describe which groupings were chosen and why	6	Line 105-106
Statistical methods	12	(*a*) Describe all statistical methods, including those used to control for confounding	6-7	Line 109-137
		(*b*) Describe any methods used to examine subgroups and interactions	6	Line 109-120
		(*c*) Explain how missing data were addressed	5	Line 93-96
		(*d*) *Cohort study*—If applicable, explain how loss to follow-up was addressed<br><br>*Case-control study*—If applicable, explain how matching of cases and controls was addressed<br><br>*Cross-sectional study*—If applicable, describe analytical methods taking account of sampling strategy	not applicable	
		(*e*) Describe any sensitivity analyses	6-7	Line 122-137
**Results**				
Participants	13*	(a) Report numbers of individuals at each stage of study—eg numbers potentially eligible, examined for eligibility, confirmed eligible, included in the study, completing follow-up, and analysed		
		(b) Give reasons for non-participation at each stage		
		(c) Consider use of a flow diagram		
Descriptive data	14*	(a) Give characteristics of study participants (eg demographic, clinical, social) and information on exposures and potential confounders		
		(b) Indicate number of participants with missing data for each variable of interest		
		(c) *Cohort study*—Summarise follow-up time (eg, average and total amount)		
Outcome data	15*	*Cohort study*—Report numbers of outcome events or summary measures over time		
		Case-control study—Report numbers in each exposure category, or summary measures of exposure		
		Cross-sectional study—Report numbers of outcome events or summary measures		

Main results	16	(*a*) Give unadjusted estimates and, if applicable, confounder-adjusted estimates and their precision (eg, 95% confidence interval). Make clear which confounders were adjusted for and why they were included	8-9	163-182
		(*b*) Report category boundaries when continuous variables were categorized	9	185-192
		(*c*) If relevant, consider translating estimates of relative risk into absolute risk for a meaningful time period	not applicable	
Other analyses	17	Report other analyses done—eg analyses of subgroups and interactions, and sensitivity analyses	9-10	Line 184-201
**Discussion**				
Key results	18	Summarise key results with reference to study objectives	10	Line 205-212
Limitations	19	Discuss limitations of the study, taking into account sources of potential bias or imprecision. Discuss both direction and magnitude of any potential bias	13-14	Line 270-288
Interpretation	20	Give a cautious overall interpretation of results considering objectives, limitations, multiplicity of analyses, results from similar studies, and other relevant evidence	10-12	Line 214-257
Generalisability	21	Discuss the generalisability (external validity) of the study results	14	Line 291-300
**Other information**				
Funding	22	Give the source of funding and the role of the funders for the present study and, if applicable, for the original study on which the present article is based	15	Line 306-308

***Give information separately for cases and controls in case-control studies and, if applicable, for exposed and unexposed groups in cohort and cross-sectional studies.**
